# Percutaneous Mitral Balloon Commissurotomy in an African Patient With Left‐Sided Inferior Vena Cava: A Rare Case Report

**DOI:** 10.1155/cric/1699533

**Published:** 2026-06-30

**Authors:** Mohammed Bedru Sebah, Yidnekachew Asrat Birhan, Gashaw Solela

**Affiliations:** ^1^ Division of Cardiology, Department of Internal Medicine, Cardiac Center of Ethiopia and Saint Paul′s Millennium Medical College, Addis Ababa, Ethiopia; ^2^ Division of Cardiology, Department of Internal Medicine, College of Health Sciences, Addis Ababa University, Addis Ababa, Ethiopia, aau.edu.et

**Keywords:** left-sided inferior vena cava, percutaneous mitral balloon commissurotomy, rheumatic mitral stenosis, transposition of inferior vena cava

## Abstract

This case report describes a rare clinical scenario involving a 40‐year‐old Ethiopian female patient with very severe rheumatic mitral stenosis, found to have an anomalous transposition of the inferior vena cava (IVC). She successfully underwent a technically challenging percutaneous mitral balloon commissurotomy. To the best of our knowledge, this is the first documented case in an African patient where such a procedure has been successfully performed in the context of a congenital IVC anomaly.

## 1. Introduction

Chronic rheumatic valvular heart disease remains a major public health concern, particularly in developing countries. Ethiopia has one of the highest reported prevalences of rheumatic heart disease globally, affecting approximately 19 per 1000 school‐aged children [1]. For patients with favorable valve morphology and isolated mitral stenosis, percutaneous mitral balloon commissurotomy (PMBC) is considered the first‐line treatment, offering excellent short‐ and long‐term outcomes when performed in experienced centers [2, 3].

Anomalies or transpositions of the inferior vena cava (IVC) are rare, occurring in approximately 0.2%–0.5% of the general population [4, 5]. These anomalies pose technical challenges during catheter‐based procedures due to the aberrant venous course, which typically includes a double curvature at the iliac bifurcation and a sharp anterior angulation at the infrahepatic level [6].

This report presents the first documented case of a 40‐year‐old Black African female patient with very severe rheumatic mitral stenosis and a transposed IVC who successfully underwent PMBC, highlighting both the anatomical complexity and the feasibility of intervention in resource‐limited settings.

## 2. Case Presentation

A 40‐year‐old Black Ethiopian woman with chronic rheumatic valvular heart disease and atrial fibrillation presented with a five‐year history of gradually worsening exertional dyspnea, fatigue, and episodic palpitations. Her symptoms had progressed from limiting routine household activities to interfering with daily functioning. She had been receiving warfarin 2.5 mg, digoxin 0.125 mg, atenolol 50 mg, and furosemide 20 mg once daily with reported good adherence. Despite this regimen, she noted declining exercise tolerance and increasing exertional symptoms, prompting further evaluation.

On physical examination, the patient was comfortable at rest. Her vital signs were notable for a blood pressure of 112/64 mmHg, pulse of 83 beats per min with an irregularly irregular rhythm, respiratory rate of 20 breaths per min, and oxygen saturation of 93% on room air. She was 164 cm tall, weighed 55 kg, and had a body mass index of 20 kg/m^2^. Cardiac auscultation revealed a low‐pitched, rumbling diastolic murmur best heard at the apex, accompanied by an accentuated P2. There were no clinical signs of volume overload, including raised jugular venous pressure, S3 gallop, hepatomegaly, or peripheral edema. Examination of the chest, lungs, abdomen, and other systems was unremarkable.

Laboratory investigations revealed a white blood cell count of 4.1 × 10^3^/*μ*L (reference range: 3.9–10 × 10^3^), hemoglobin 15.5 g/dL (11.2–15.7), and platelet count 202 × 10^3^/*μ*L (182–369), all within normal limits. Serum creatinine was 0.76 mg/dL (0.59–1.04), indicating preserved renal function. Her international normalized ratio (INR) was 2.99, consistent with therapeutic anticoagulation. Serologic tests for Hepatitis B surface antigen, Hepatitis C antibody, and human immunodeficiency virus were negative.

**Figure 1:**
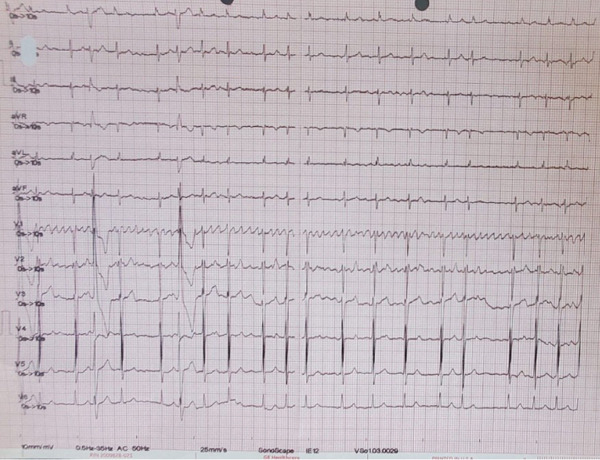
The 12‐lead ECG demonstrated atrial fibrillation with a heart rate of 112 beats per min, characterized by an irregularly irregular ventricular rhythm, absence of discrete *P* waves, and variable R–R intervals. Occasional premature ventricular complexes were also noted.

The chest radiograph demonstrated cardiomegaly with straightening of the left heart border, convexity of the right heart border, and features suggestive of pulmonary hypertension. Electrocardiography revealed atrial fibrillation with a heart rate of 112 beats per min as shown in Figure 1.

**Figure 2:**
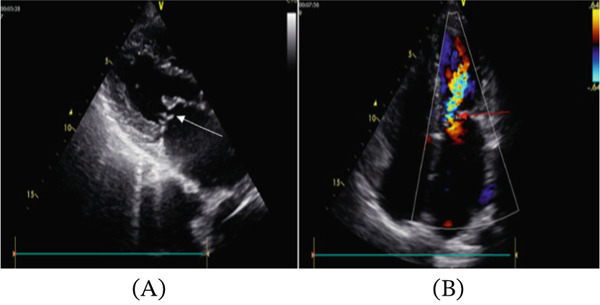
(A) Parasternal long‐axis view showing markedly thickened mitral valve leaflets with restricted diastolic opening (white arrow), consistent with severe rheumatic mitral stenosis. (B) Apical four‐chamber view with color Doppler demonstrating turbulent flow across the mitral valve (red arrow), indicating significant transmitral gradient.

Transthoracic echocardiography (Figure 2) demonstrated very severe mitral stenosis with a mitral valve area of 0.9 cm^2^, severe pulmonary hypertension with an estimated pulmonary artery systolic pressure of 90 mmHg, and favorable valve morphology for PMBC, with a Wilkins score of 7/16 [7].

## 3. Procedure and Immediate Outcome

**Figure 3:**
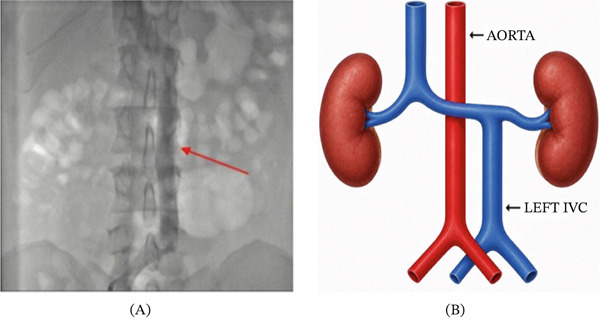
(A) Sheath venogram from the right common femoral vein demonstrating a left‐sided inferior vena cava (IVC), indicated with a red arrow, terminating at the left renal vein and crossing anterior to the aorta to join the normal prehepatic segment of the IVC. (B) Schematic diagram demonstrating a left‐sided IVC (blue) ascending on the left, draining into the left renal vein, and crossing anterior to the abdominal aorta (red) to connect with the normal right‐sided prehepatic IVC.

Prior to the planned PMBC, venography unexpectedly demonstrated a left‐sided IVC (Figure 3A), adding anatomical complexity to the intervention. The procedure was carried out under strict aseptic conditions in the cardiac catheterization laboratory via the right femoral vein, with the patient under local anesthesia. Prophylactic antibiotics were administered. Transseptal puncture was carried out using the Brockenbrough technique [8]. Following femoral venous access, an 8.5 French Mullins sheath was advanced over a 0.032‐inch exchange‐length guidewire into the superior vena cava.

During this process, an abnormal trajectory of the wire was observed, prompting contrast injection, which revealed a left‐sided transposition of the IVC, indicated by the red arrow in Figure 3A and illustrated in the schematic diagram in Figure 3B. Despite this vascular anomaly, the procedure proceeded with cautious manipulation.

**Figure 4:**
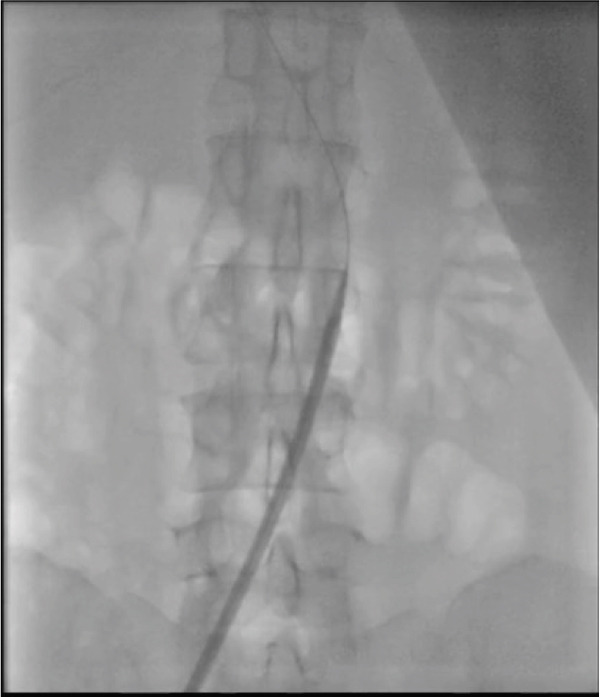
Fluoroscopic image demonstrating advancement of the Mullins dilator over the coiled guidewire through the left‐sided inferior vena cava during PMBC.

**Figure 5:**
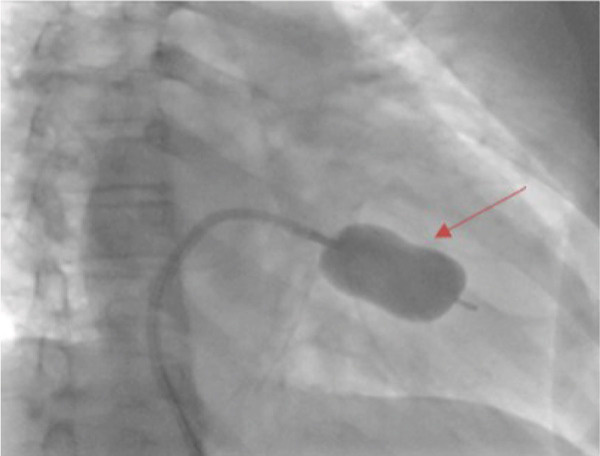
Fluoroscopic image of the Accura balloon, fully inflated across the mitral valve during percutaneous mitral balloon commissurotomy (PMBC), indicated by the red arrow.

The Mullins dilator over the coiled guidewire (Figure 4) and balloon assembly were successfully advanced through the left‐sided IVC into the left atrium. PMBC was subsequently performed using a 26‐mm Accura balloon, with sequential inflations to 24 mm followed by 25 mm (Figure 5). The procedure was completed without complications. Postprocedural hemodynamic measurements are summarized in Table 1, demonstrating a favorable immediate outcome.

**Table 1:** Pre and postprocedure valve area and hemodynamic parameters.

Parameter	Pre‐PMBC	Post‐PMBC
MVA ^∗^	0.9 cm^2^	2 cm^2^
MR	Nil	Nil
LA mean pressure	31 mmHg	16 mmHg
MV mean gradient	22 mmHg	7 mmHg
PASP	90 mmHg	60 mmHg

*Note:*  ^∗^ By planimetry.

Abbreviations: LA, left atrium; MR, mitral regurgitation; MV, mitral valve; MVA, mitral valve area; PASP, pulmonary artery systolic pressure; PMBC, percutaneous mitral balloon commissurotomy.

## 4. Follow‐Up and Long‐Term Outcome

During more than 18 months of outpatient follow‐up, which included regular clinical assessments and serial echocardiography at intervals of 3–6 months, the patient remained asymptomatic, required no diuretics, and experienced no heart failure–related hospitalizations. Echocardiography at 18 months showed a mitral valve area and transmitral pressure gradient comparable to the immediate postprocedural measurements, reflecting sustained procedural success. She continued warfarin 5 mg daily, digoxin 0.125 mg daily, and atenolol 50 mg daily, maintaining a therapeutic INR and well‐controlled heart rate throughout follow‐up.

## 5. Discussion

PMBC is the treatment of choice for patients with severe symptomatic mitral stenosis and favorable valve morphology [2, 3]. This intervention is generally contraindicated in the presence of more than mild mitral regurgitation or left atrial thrombus. The Wilkins scoring system, which assesses leaflet mobility, thickening, calcification, and subvalvular involvement, is commonly used to determine suitability for PMBC; a score of ≤ 8 typically predicts favorable outcomes [7].

The Inoue technique, which involves accessing the left atrium via the right femoral vein and transseptal puncture, remains the most widely adopted approach [9]. However, anatomical variations of the IVC, such as left‐sided IVC, can pose significant technical challenges during transfemoral procedures [10, 11].

Left‐sided IVC is a rare congenital anomaly resulting from persistence of the left supracardinal vein with regression of the right supracardinal vein during embryologic development. Although usually asymptomatic, this variant alters the typical anatomical route of venous return, complicating catheter manipulation due to the vessel′s double curvature at the iliac bifurcation and anterior course near the infrahepatic segment. These features can hinder the advancement of sheaths and devices during percutaneous cardiac interventions [6, 10, 12].

Several case reports have documented adaptations to standard PMBC techniques in patients with anomalous IVC anatomy, including access via the left femoral vein, transjugular route, transhepatic access, and even retrograde nontransseptal approaches. For example, PMBC has been successfully performed via a left transhepatic vein in a 42‐year‐old woman with dextrocardia, situs inversus totalis, and IVC interruption. Another report described balloon mitral valvuloplasty (BMV) through the left femoral vein in a patient with an inaccessible right femoral vein and anomalous IVC course. In one notable case, an incidentally discovered IVC occlusion was treated with balloon dilation, followed by successful PBMV using the Inoue technique[12–14].

In our patient, fluoroscopic guidance was used to carefully navigate a specially angled catheter through the right femoral vein and anomalous left‐sided IVC into the right atrium. After successful transseptal puncture, the Inoue balloon was advanced to the mitral valve, and PMBC was performed without complication. The postprocedural mitral valve area increased significantly, with marked symptomatic improvement.

To the best of our knowledge, this represents the first documented case in Africa of a successful PMBC performed via the right femoral vein in a patient with rheumatic mitral stenosis and left‐sided IVC. This case underscores the importance of recognizing vascular anomalies and adapting procedural techniques accordingly, especially in resource‐limited settings where advanced imaging may not be routinely available.

## 6. Conclusion

To our knowledge, this is the first reported case of a successful PMBC in an African patient with a left‐sided IVC. This case underscores the procedural challenges posed by rare venous anomalies and highlights the critical importance of thorough preprocedural assessment, anatomical awareness, and technical adaptability in transcatheter structural heart interventions—particularly in resource‐limited settings.

## Author Contributions

M.B.S. was involved in conceptualization, data curation, validation, writing—original draft, and writing—review and editing. Y.A.B. and G.S. were involved in data curation, validation, writing—review and editing.

## Funding

No funding was received for this manuscript.

## Disclosure

All authors read and approved the final manuscript.

## Ethics Statement

The authors have nothing to report.

## Consent

Written informed consent was obtained from the patient for publication of this case report and any accompanying images. A copy of the written consent is available for review by the editor‐in‐chief of this journal.

## Conflicts of Interest

The authors declare no conflicts of interest.

## Data Availability

Data supporting this case report will be available with the corresponding author upon reasonable request.
